# Predicting exercise intolerance in elderly individuals with heart failure using the 30-second chair stand test

**DOI:** 10.1016/j.ijcha.2024.101464

**Published:** 2024-07-10

**Authors:** Marco Savino, Luigi Savino, Pasquale Mone, Concetta Schiano, Antonio De Luca, Gaetano Santulli

**Affiliations:** a Department of Medicine, Division of Cardiology, Wilf Family Cardiovascular Research Institute, Einstein – Mount Sinai Diabetes Research Center *(ES-DRC)*, Einstein Institute for Neuroimmunology and Inflammation *(INI)*, Albert Einstein College of Medicine, New York City, NY, USA; b Department of Medicine and Health Sciences “*Vincenzo Tiberio*”, University of Molise, Campobasso, Italy; c Casa di Cura *Montevergine*, Mercogliano (Avellino), Italy; d Department of Advanced Medical and Surgical Sciences (*DAMSS*) University of Campania "*Luigi Vanvitelli*", Naples, Italy; e Department of Mental and Physical Health and Preventive Medicine, University of Campania "*Luigi Vanvitelli*", Naples, Italy; f International Translational Research and Medical Education (*ITME*) Consortium, Academic Research Unit, and Department of Advanced Biomedical Sciences, “*Federico II*” University, Naples, Italy; g Department of Molecular Pharmacology, Einstein Institute for Aging Research, Fleischer Institute for Diabetes and Metabolism (*FIDAM*), Albert Einstein College of Medicine, New York City, NY, USA

**Keywords:** 30CST, Aging, CPX, Elderly, Exercise, HF, Physical activity

Heart failure (HF) is a clinical syndrome associated with high rates of morbidity and mortality. Without treatment, HF can progress through four stages of the disease (A-D). The American College of Cardiology Foundation/American Heart Association defines the stages of HF as follows: Stage A includes people at high risk for developing HF but without structural heart disease or symptoms; Stage B involves individuals with structural heart disease but no obvious signs or symptoms of HF; Stage C encompasses patients with structural heart disease who have had previous or current HF symptoms; and Stage D refers to refractory HF.

HF represents an increasing concern especially in the senior population. A variety of methods can be used to identify older patients with HF and exercise intolerance, which is one of the most important parameters to evaluate the progression from A/B HF stage to C/D HF stage. Exercise intolerance, indicated by dyspnea and fatigue during exertion, is a cardinal manifestation of HF [1]. Exercise intolerance is defined as an impaired ability to perform physical activity and to reach the expected age-related level of exercise duration. The gold standard to assess exercise tolerance is the Cardiopulmonary exercise test (CPX), which allows us to estimate the peak oxygen uptake (peak VO₂). However, this method, although effective, is not always practical due to its expense and equipment needs [2]. There is therefore a need for simpler and easily accessible tools that are equally reliable. To fill this gap, in this issue of *IJC Heart & Vasculature*, Kobayashi and collaborators [3] investigated the potential value of an alternative approach known as “The 30-second chair stand (30CS) test” as a predictor of exercise tolerance in elderly individuals with stage A/B heart failure. The 30CS measures the number of sit-to-stand repetitions performed in 30 s [4], [5], [6]. The goal of the study was to identify a clear cut-off point of low exercise tolerance to distinguish patients. This methodology is also broadly used to evaluate exercise tolerance in patients suffering from different diseases [7], [8]. The Authors designed a single-center, cross-sectional, observational study, in which the use of the 30CS was explored as an alternative to CPX. The authors enrolled 493 (296 males and 197 females) elderly outpatients (≥75 years) and measured their exercise tolerance using both the 30CS and CPX. Of note, patients with pulmonary disease were not excluded from the study.

Patients were evaluated considering: demographic and clinical characteristics (age, sex, presence or absence of heart disease, diabetes, dyslipidemia), frailty (using the Kihon checklist, KCL, a binary self −administered questionnaire comprising 25 items across seven domains: nutrition, oral health, instrumental activities of daily living, confinement, exercise, cognition, and depression [9]), body composition through bioelectrical impedance analysis, exercise habits determined using the five-item Stage of Change for Exercise Behavior (SOC), motor function (investigated via three parameters: grip strength, 30CS, and one-leg standing time), exercise tolerance (measured using a bicycle ergometer with a progressively increasing load). The following findings emerged (Fig. 1): male participants achieved a mean 30CS score of 20.3 ± 5.4 times, while female participants achieved a mean 30CS score of 19.1 ± 5.0 times. The mean peak VO₂ was 21.4 ± 4.2 and 18.0 ± 3.7 mL/min/kg for males and females, respectively [3]. The results revealed a significant association between 30CS scores and peak VO₂ values, a gold standard indicator of exercise tolerance. Specifically, after adjustment, every 1-unit increase in peak VO_2_ was associated with a 0.255 and 0,282 unit increase in 30CS, in males and females, respectively. The cut-off values of 30CS were established as 18 times for males and 16 times for females.

**Fig. 1:**
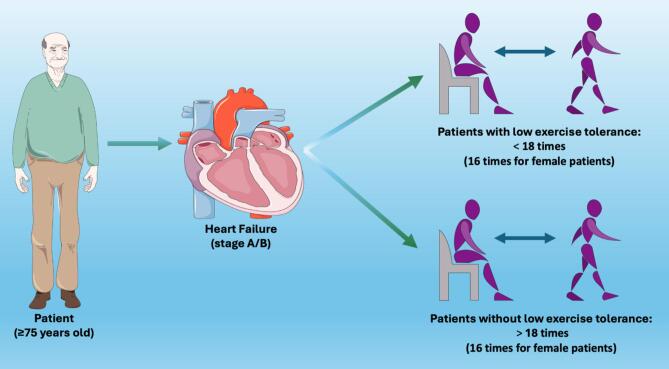
Determination of a cut-off of 18 times in the 30-second chair stand test to distinguish patients with or without exercise tolerance in a population of elderly patients with stage A/B heart failure.

This work lays the foundation for the use of the 30CS, which is simpler to perform and more economical, for the evaluation of elderly and frail patients with stage A/B HF in relation to exercise tolerance and HF progression. However, the study also presents some limitations that need to be highlighted; among these, the most important could be the measurement of heart rate and blood pressure at rest and during exercise together with the measurement of peak VO_2_ maximum during exercise. Furthermore, the 30CS test was administered only once in each patient. Previous observations have shown that differences in temporal patterns of 30CS performance corresponded to differences in certain dimensions of physical and mental function [10]. Additionally, it could be useful to differentiate between patients in stage A and stage B of HF, to be able to evaluate if/how the results of 30CS and VO_2_ peak change with the disease progression. In addition, it could be interesting to assess changes in the 30CS test in patients who are in all four stages of HF, to be able to establish and validate cut-offs for each of the four stages.

## Declaration of competing interest

The authors declare that they have no known competing financial interests or personal relationships that could have appeared to influence the work reported in this paper.
